# In Vitro and In Silico Characterization of an Antimalarial Compound with Antitumor Activity Targeting Human DNA Topoisomerase IB

**DOI:** 10.3390/ijms22147455

**Published:** 2021-07-12

**Authors:** Bini Chhetri Soren, Jagadish Babu Dasari, Alessio Ottaviani, Beatrice Messina, Giada Andreotti, Alice Romeo, Federico Iacovelli, Mattia Falconi, Alessandro Desideri, Paola Fiorani

**Affiliations:** 1 Department of Biology, University of Rome Tor Vergata, Via della Ricerca Scientifica 1, 00133 Rome, Italy; binichhetri.14@gmail.com (B.C.S.); jagadishdrdasari@gmail.com (J.B.D.); beatrice.messina702@gmail.com (B.M.); giada0404@gmail.com (G.A.); alice.romeo@uniroma2.it (A.R.); federico.iacovelli@uniroma2.it (F.I.); falconi@uniroma2.it (M.F.); desideri@uniroma2.it (A.D.); paola.fiorani@uniroma2.it (P.F.); 2 Institute of Translational Pharmacology, National Research Council, CNR, Via del Fosso del Cavaliere 100, 00133 Rome, Italy

**Keywords:** human DNA topoisomerase, cancer, drug, molecular docking

## Abstract

Human DNA topoisomerase IB controls the topological state of supercoiled DNA through a complex catalytic cycle that consists of cleavage and religation reactions, allowing the progression of fundamental DNA metabolism. The catalytic steps of human DNA topoisomerase IB were analyzed in the presence of a drug, obtained by the open-access drug bank Medicines for Malaria Venture. The experiments indicate that the compound strongly and irreversibly inhibits the cleavage step of the enzyme reaction and reduces the cell viability of three different cancer cell lines. Molecular docking and molecular dynamics simulations suggest that the drug binds to the human DNA topoisomerase IB-DNA complex sitting inside the catalytic site of the enzyme, providing a molecular explanation for the cleavage-inhibition effect. For all these reasons, the aforementioned drug could be a possible lead compound for the development of an efficient anti-tumor molecule targeting human DNA topoisomerase IB.

## 1. Introduction

Biologically, topoisomerases play a vital role in the maintenance of genomic integrity inside the cell by controlling DNA torsional stress [1]. This is accomplished by transiently breaking and rejoining DNA strands. Human DNA topoisomerase IB (htopIB) is one of six different topoisomerases that exist in humans [2]. HtopIB is a nuclear enzyme and, with type IA topoisomerase, belongs to the topoisomerase I subfamily that cuts only one DNA strand. The type IA topoisomerases bind covalently to the 5′ end of the cleaved DNA, whereas the type IB are attached to the 3′ end during the catalytic cycle. Among topoisomerase families there are also type II topoisomerases that cut both DNA strands [2]. 

HtopIB is a monomeric 91 kDa enzyme consisting of 765 amino acids and is composed of four domains: the N-terminal domain (residues 1–214), which is never crystallized due to a high degree of flexibility and which contains nuclear localization signals; the core domain (residues 215–635) which can be further divided into subdomains I, II, and III; linker domain (residues 636–712) connecting subdomain III with the C-terminal; and the C-terminal domain (residues 713–765) containing the catalytic residue Tyr723 [3,4]. The linker domain, formed by two long helices, is involved in controlling the relaxation mechanism. Due to its shape and its positive charge, this domain interacts with the DNA downstream of the cleavage site and drives relaxation through a controlled rotation [5,6,7,8]. The enzyme relaxes both positive and negative supercoils by creating a nick on one strand of the DNA duplex, forming a transient phospho-tyrosine bond [3]. This transient bond occurs through a nucleophilic attack on DNA by Tyr723 that breaks one of the DNA strands, leaving the enzyme covalently attached to the 3′-phosphate and forming a protein–DNA covalent complex called “cleavage complex”. At this point, the cut DNA strand can rotate around the other one, changing the linking number and consequently the DNA topology. The htopIB catalytic cycle can be summarized in five steps: (1) non-covalent DNA binding; (2) nucleophilic attack by the Tyr723 residue; (3) strand rotation of the nicked strand around the intact strand to relax the supercoiled DNA; (4) religation of the DNA strand; and (5) enzyme release [4].

HtopIB is the unique target of a class of anticancer compounds belonging to the camptothecin (CPT) family [9,10]. CPT, a pentacyclic alkaloid extracted from the plant *Camptotheca acuminata*, is an E-ring lactone that is poorly soluble and toxic for the organism, but a series of more soluble derivatives have been developed [11] and two of them, topotecan (TPT) and irinotecan, are in clinical use as a second line therapy for ovarian, lung, and colorectal cancers [12,13,14]. Once the enzyme has cut a supercoiled DNA to form the protein–DNA complex, CPT binds to this transient complex by intercalating in the cleavage site and slowing down the religation step [15,16,17]. The increased lifetime of the covalent complex leads to the collapse of the replicative fork with the formation of double strand breaks and, if not repaired, consequent cell death. The CPT inhibition is called a poison because it causes the stalling of the protein on DNA that leads to cell death. Other htopoIB poisons are the indenoisoquinolines which the planar polycyclic cores allow the intercalation of at the strand breakage site in a CPT-like manner. However, indenoisoquinolines are less reversible than CPT and thus their pharmacokinetics enable the formation of persistent cleavage complexes and potential shorter infusion times [18]. Besides poisons, other types of htopIB inhibitors are catalytic inhibitors that act by preventing the enzyme’s binding to DNA or cleavage step. Among them we can count erybraedin C, which is a cleavage inhibitor [19], or benzoxazines, such as 1,4-benzoxazin-3-ones and 2,4-Dihydroxy-1,4-benzoxazin-3-one, which prevent enzyme binding to DNA [11]. Erybraedin C contains a tetracyclic ring system and is characterized by the presence of two hydroxy groups and two prenyl groups [19], while benzoxazines are a group of heterocyclic chemical compounds that consist of a benzene ring fused to an oxazine ring [11]. 

DNA topoisomerases have been shown to be a promising therapeutic target not only against cancer but also against bacteria and parasites [20,21,22]. An example is the topoisomerase from apicomplexan parasites, like *Plasmodium falciparum*, which may be a striking target for drug development [23,24]. 

Open-access drug banks are an interesting source of compounds for a wide range of different diseases since, they contain hundreds of drugs for which a target is still unknown [25]. Among them, Pathogen Box by Medicines for Malaria Venture is a product development partnership with the aim of reducing the burden of malaria by delivering novel, efficient and reasonably priced anti-malarial drugs for disease-endemic countries.

The Pathogen Box contains 400 different drug-like molecules active in neglected diseases (including malaria, tuberculosis, dengue, and kinetoplastids). These compounds were designed as drugs for the treatment of neglected diseases, but, as history has taught us, a drug designed for a specific pathology has sometimes proved to be much more effective in treating a completely different one. For this reason, we considered that among them there could be a novel compound to be used as an anti-tumor drug. We then screened the activity of 125 compounds designed for malaria treatment against htopIB, due to the similarity of *Plasmodium falciparum* topoisomerase with its human counterpart [26].

**Figure 1:**
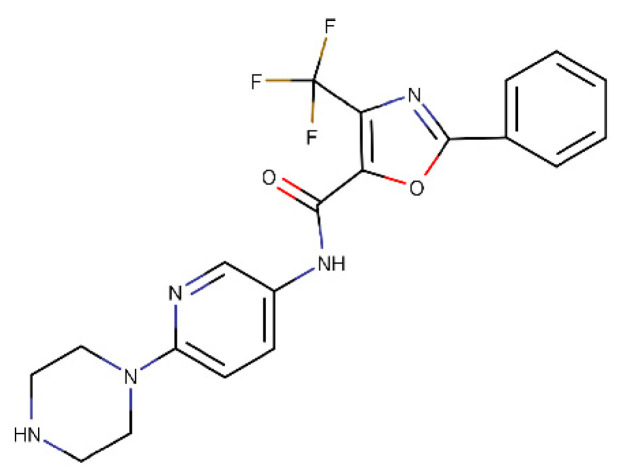
Molecular structure of MMV024937.

During the screening, we selected one compound, MMV024937 (Figure 1), which efficiently inhibits htopIB.

In this paper we investigated the effect of MMV024937 on the catalytical cycle of htopIB through experimental and computational approaches, and we tested its effect on three different cancer cell lines. The results indicate that the compound inhibits the htopIB cleavage activity in vitro by binding to the enzyme’s active site. Furthermore, it strongly reduced cell viability at concentrations between 50 and 100 µM. 

## 2. Results

### 2.1. MMV024937 Inhibits the Catalytic Activity of htopIB

**Figure 2:**
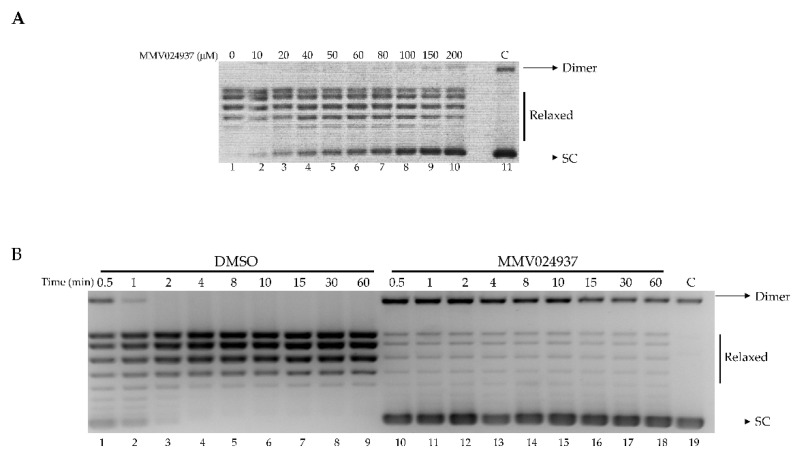
Relaxation of supercoiled DNA. (**A**) Relaxation of a negatively supercoiled plasmid DNA by htopIB at increasing concentrations of MMV024937 (lanes 2–10); lane 1: no drug added and lane 11: no protein added. (**B**) Relaxation of negative supercoiled plasmid DNA in a time course experiment with DMSO (lanes 1–9), 100 μM MMV024937 dissolved in DMSO (lanes 10–18), lane 19, no protein added. The reaction products are resolved on agarose gel and visualized with ethidium bromide. Dimer indicates dimer supercoiled plasmid DNA; SC indicates supercoiled plasmid DNA.

The inhibitory effect of MMV024937 on htopIB activity was assessed by a plasmid relaxation assay (Figure 2A). Wild-type protein was incubated with a supercoiled plasmid in the absence or presence of increasing concentrations of MMV024937, and the relaxation activity was monitored after 1 h. The results indicated that MMV024937 inhibited the relaxation activity of htopIB in a dose-dependent manner (Figure 2A). Addition of MMV024937 to the enzyme and DNA determined an inhibition of the relaxation activity that was already observable at a concentration of 20 μM (Figure 2A, lane 3) and became maximal at a concentration of 150/200 μM (Figure 2A, lanes 9 and 10). Since MMV024937 is dissolved in DMSO, we evaluated the enzyme activity in the presence of an identical concentration of solvent without MMV024937, demonstrating that DMSO does not affect the relaxation activity of htopIB (Figure 2A, lane 1). In addition, the compound’s effect on the electrophoretic mobility of DNA in absence of htopIB was evaluated (Figure 2A, lane 11). 

The assay was carried out as a function of time in the presence of MMV024937 at a concentration of 100 μM. This experiment showed that the inhibitory effect is maintained over time (Figure 2B, lanes 10–18), to minimally 1 h, indicating an irreversible inhibition of the enzyme’s catalytic activity. As a control, the relaxation assay was performed in the presence of DMSO alone (Figure 2B, lanes 1–9).

### 2.2. Cleavage with CL14/CP25 Suicide Substrate

**Figure 3:**
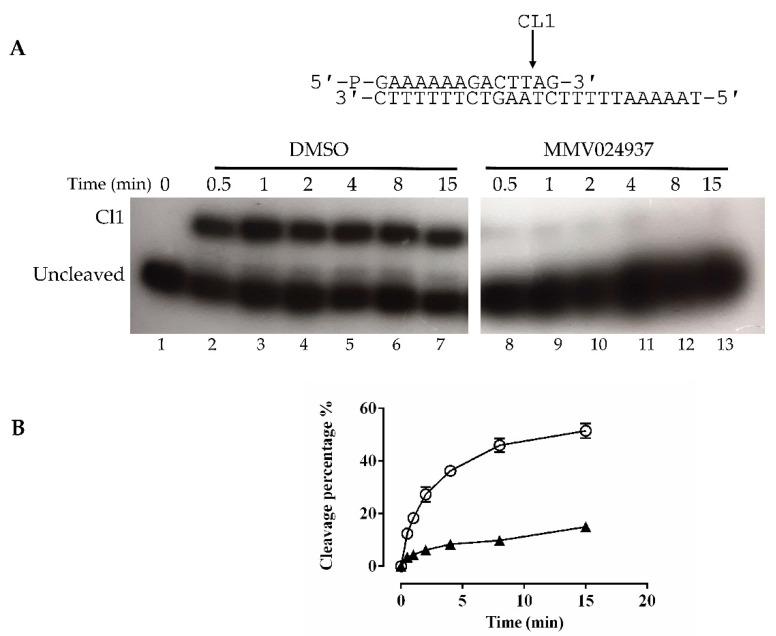
Cleavage kinetics. (**A**) Cleavage reaction of the enzyme with the CL14/CP25 suicide substrate, shown at the top of the figure, in a time course experiment in the presence of only DMSO (lanes 2–7), or MMV024937 dissolved in DMSO (lanes 8–13). In lane 1 the protein was not added. CL1 represents the DNA strand cleaved by the enzymes at the preferred cleavage site, indicated by an arrow. (**B**) Percentage of cleaved suicide substrate, normalized to the total amount of each lane, plotted against time for the reaction with only DMSO (circles) and MMV024937 dissolved in DMSO (triangles). Data are means ± SD obtained from three independent experiments.

The cleavage activity of the htopIB was analyzed in a time course experiment using a CL14/CP25 suicide cleavage substrate. In detail, a 5′-end radiolabeled oligonucleotide CL14 (5′-GAAAAAAGACTTAG-3′) was annealed to the CP25 (5′-TAAAAATTTTTCTAAGTCTTTTTTC-3′) complementary strand to produce a duplex with an 11 base 5′ single-strand overhang (Figure 3A). 

In this experiment the religation step was precluded because the AG-3′ dinucleotide is too short to be religated, leaving the enzyme covalently attached to the 12 oligonucleotide 3′-end. Three units of wild-type enzyme were incubated in the absence and presence of MMV024937, and the reactions were stopped at increasing time intervals ranging from 0.5 min to 15 min. Samples were precipitated by using 100% ethanol, then digested by trypsin, and the products were resolved on a denaturing urea polyacrylamide gel (Figure 3A). Figure 3A shows that in the presence of MMV024937 the cleavage does not occur at all (lanes 8–13), while the control, in the presence of DMSO, showed a typical cleavage kinetics (Figure 3A, lanes 2–7). The percentage of the cleaved fragment (CL1), normalized to the total radioactivity in each lane plotted against time for DMSO (Figure 3B, circle) and MMV024937 incubation (Figure 3B, triangle), confirmed the full inhibitory effect of the MMV024937 compound.

### 2.3. Cell Viability Assay

**Figure 4:**
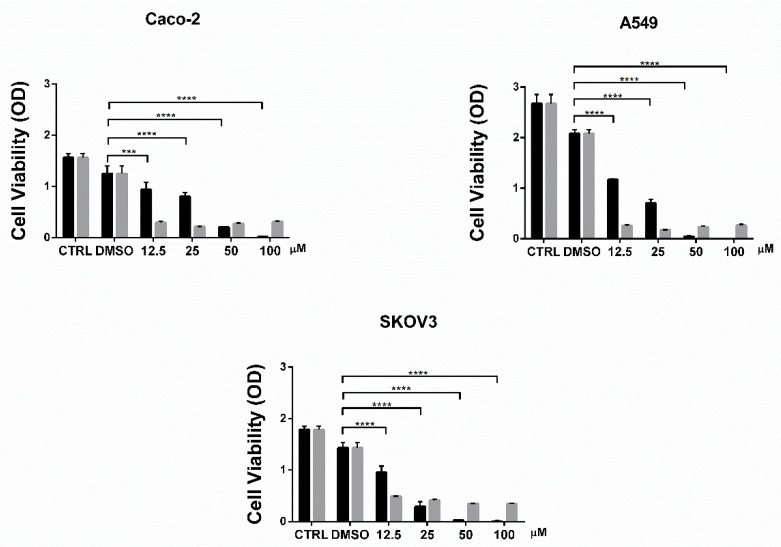
Cell viability assay in the presence of MMV024937 and TPT. Cytotoxicity of MMV024937 (black) and TPT (gray) were tested on three different cancer cell lines; Caco-2 (top left panel), A-549 (top right panel), and SKOV-3 (bottom panel). The figure reports cumulative data analyzed by a two-way ANOVA test, with mean ±SD values. **** *p* < 0.0001 and *** *p* < 0.001.

The cytotoxic effect of MMV024937 on the colon carcinoma cancer cell line (Caco-2), non-small cell lung cancer cell line (A-549), and ovarian cancer cell line (SKOV-3) was evaluated via an MTT assay in comparison to TPT. The drugs were tested at various concentrations, ranging from 12.5 μM to 100 μM in a 96-well plate, containing 10^4^ cells/well. The cells were incubated in the presence of various concentrations of the drug, or in the presence of the same amount of DMSO as a control, for 48 h at 37 °C under 5% CO_2_ and 95% air. After the incubation, the medium was removed and 200 μL of fresh media supplemented with MTT reagent at a final concentration of 0.5 mg/mL was added for 4 h. The reagent was then replaced with 100 μL DMSO and incubated at room temperature, covered from light, on a shaking plate for 15 min, and the absorbance was measured at 570 nm using a microplate reader. The MMV024937 compound affected cell viability in a dose-dependent manner with an inhibition, at 50 and 100 μM stronger than TPT, which displays a constant inhibition from 12.5 to 100 μM (Figure 4). The compound exhibited a larger cytotoxic effect on the SKOV-3 cell line, where cell viability started to decrease at 25 μM, indicating that MMV024937 had a cell line-dependent effect (Figure 4).

### 2.4. Molecular Docking Simulations

Molecular docking simulations were performed to structurally evaluate the interaction of MMV024937 with a DNA-bound htopIB structure (PDBID: 1T8I) [27], mimicking the experimental conditions of simultaneous incubation of the enzyme, the drug, and the DNA. All the molecular docking simulations performed indicated that the drug interacts with the htopIB-DNA complex with a high average energy of about −12.0 kcal/mol, achieving binding at two preferential locations within the htopIB-DNA binding cavity. 

**Figure 5:**
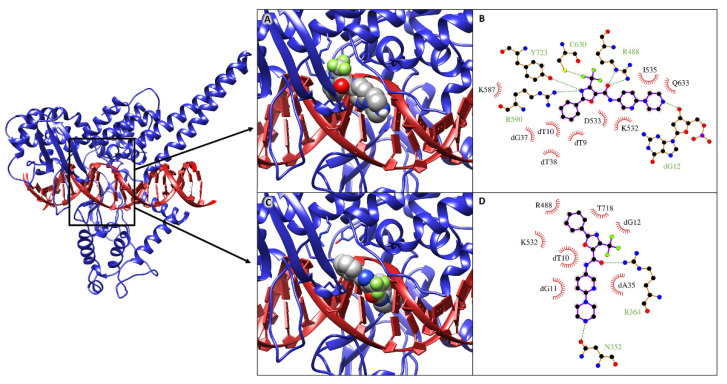
On the left: 3D structure of the htopIB-DNA complex (PDBID: 1T8I), used as receptor for the molecular docking simulations. The binding site is highlighted by a rectangular box. The htopIB structure is represented as a blue cartoon, while the DNA is shown as a red ribbon. (**A**,**C**) 3D best binding poses obtained for the drug MMV024937 in “non intercalated” (**A**) and “intercalated” (**C**) configuration. The MMV024937 compound is shown in a space fill model, colored by atom type, with carbon atoms in grey, while htopIB catalytic residues are shown as stick models, colored by atom type, with carbon atoms in blue. The pictures were obtained using the Chimera program [28]). (**B**,**D**) 2D schematic view of the interactions between the drug and the htopIB-DNA complex in “non intercalated” (**B**) and “intercalated” (**D**) configuration. Hydrogen bonds between the drug molecule and the interacting residue/base are shown as dashed lines. Hydrophobic interactions are shown as radial half-circles. Labels indicate base or residue names in one-letter code and their numbering in the structure. The pictures were created using the LigPlot+ 1.4 software [29].

In most of the obtained binding poses the drug localizes at the center of the cavity, inserted among the catalytic pentad of the enzyme. Figure 5A represents the best binding pose, named “non-intercalated configuration”, that shows an interaction energy of −12.4 kcal/mol. In this pose the drug establishes five hydrogen bonds and five hydrophobic interactions with the surrounding residues (Figure 5B). 

In particular, the oxazole-5-carboxamide group and the trifluoromethyl group of MMV024937 insert within the catalytic site of the enzyme (Figure 5A), establishing hydrogen bonds with Arg488, Arg590, and Tyr723 and setting up hydrophobic interactions with Lys532 (Figure 5B). The drug also contacts the nearest DNA major groove, establishing four hydrophobic contacts and one hydrogen bond with the underlying nucleotides (Figure 5A,B). In the remaining binding poses, the drug intercalates within the structural distortion generated between the dT10 and dG11 nucleotides after htopIB binding. Figure 5C shows the best binding pose, named “intercalated configuration”, which reaches an interaction energy of −11.7 kcal/mol. The interaction pattern evaluated for this pose highlights two hydrogen bonds and three hydrophobic interactions with surrounding residues, in particular with Arg488 and Lys532 of the catalytic site, and four hydrophobic interactions with the nucleotides located below (Figure 5D).

### 2.5. Molecular Dynamics Simulations and MM/GBSA Analysis

The two best complexes obtained for the non-intercalated and intercalated drug configurations were further analyzed by performing 100 ns of classical molecular dynamics (MD) simulations to further validate the stability of the obtained complexes and to accurately evaluate the interaction energies between the htopIB-DNA complex and the drug. MM/GBSA analyses confirmed the strong interaction between MMV024937 and the htopIB-DNA complex, showing interaction free energies of −49.0 and −55.1 kcal/mol for the non-intercalated and intercalated drug configurations, respectively (Table 1). 

Both VdW and electrostatic interactions contribute importantly to the binding, although the electrostatic contribution is predominant in the intercalated configuration due to its close interactions with the charged DNA. MM/GBSA per-residue decomposition analyses, which allowed for estimating the contribution given by single residues or DNA bases to the total binding energy of the drug, confirmed that the drug in non-intercalated configuration closely interacts with htopIB catalytic site residues and also with some of the underlying DNA bases (Table 2). On the other hand, the intercalated drug only establishes minor contacts with the catalytic region but can strongly interact with surrounding DNA bases (Table 2). 

The high-interaction free energies indicate that MMV024937 could interfere with htopIB catalytic activity when DNA binding has already occurred, occupying the catalytic site of the enzyme and, due to the favorable electrostatic energy, altering the interactions between the negatively charged DNA and the mostly positively charged protein, which are crucial for the controlled rotation mechanism that leads to the final DNA relaxation [30].

### 2.6. Principal Component Analysis

**Figure 6:**
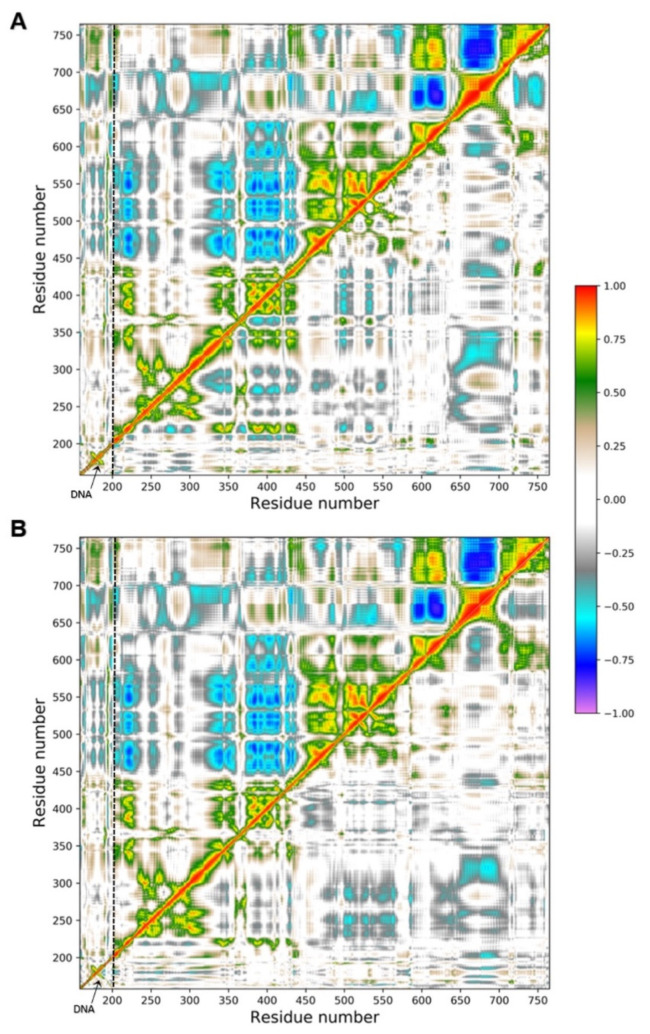
DCCMs obtained for the three simulated systems. The upper-left triangles of both graphs (**A**,**B**) represent the DCCM obtained for the unbound htopIB-DNA complex, while the lower-right triangles show the DCCMs for the non-intercalated (**A**) and intercalated (**B**) drug complexes, respectively. Color coding is reported in the legend. Positive values between two residues indicate a correlated motion, while negative values indicate an anti-correlated motion. DNA regions are indicated by labels and separated from the protein regions by dotted lines.

To better characterize the collective motions of different regions of the htopIB structure and to highlight if the presence of the drug could induce changes in the structural dynamics of the htopIB-DNA complexes, dynamic cross-correlation maps (DCCM), based on the atomic fluctuations of 565 Cα atoms of htopIB and on the 42 P atoms of DNA, were generated for each MD trajectory. Positive values indicate that the motion between two residues/bases is correlated, with residues/bases moving in the same direction, while negative values represent an anti-correlated motion, with residues/bases moving in opposite directions. A 100 ns MD simulation of the htopIB-DNA complex in the unbound state was also performed and analyzed as reference. MD simulations indicate remarkable differences between the unbound and bound complexes, and highlight the presence of functional structural motions in the htopIB unbound structure (Figure 6A,B).

The unbound htopIB-DNA simulation is characterized by highly anti-correlated motions between subdomain I (residues 215–232 and 320–433) and subdomain III (residues 450–635) of the htopIB core domain (Figure 6A,B; upper-left triangles). Strong anti-correlated motions are also present between the end region of subdomain III (residues 575–635) and the linker region (residues 650–700), and between the linker region and the htopIB C-terminal domain (residues 715–765). Similarly, correlated motions can be sparsely observed between the subdomains I, II and III (residues 200–635), and between subdomain III (residues 575–635) and the C-terminal domain (residues 715–765). As previously reported, correlated internal motions represent an important feature of the DNA-bound htopIB structure and highlight the important role played by protein-protein domain communications and conformational changes in the functional processes of this enzyme [16]. Subdomain III (residues 450–635), containing four of the five htopIB catalytic residues, the linker (residues 650–700), and the C-terminal regions also show mild positively and negatively correlated motions with the DNA region (Figure 6A,B; upper-left triangles). 

The presence of the drug within the binding pocket completely abolishes these correlated motions in both the non-intercalated and intercalated binding configurations (Figure 6A,B; lower-right triangles). In fact, in the presence of the drug, DNA motions and intra- and inter-domain protein motions become completely uncorrelated, except for a small anti-correlated movement between subdomain I (residues 300–350) and the linker region (residues 636–712), and sparse positively and negatively correlated motions within the three subdomains of the htopIB core region (residues 250–575), which was observed in both binding configurations. 

**Figure 7:**
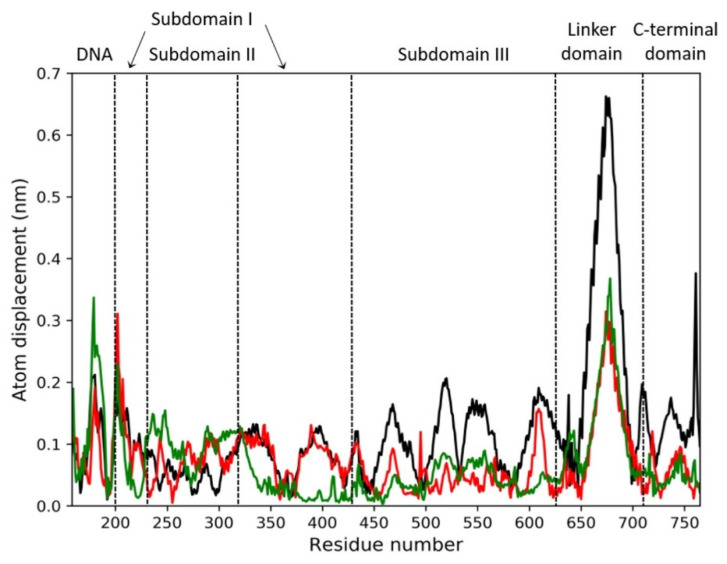
Atomic displacement calculated for each of the 565 C-alpha atoms of htopIB and each of the 42 P atoms of DNA, computed along the direction of the first eigenvector for the three MD trajectories. The black line indicates the unbound htopIB-DNA complex, while the red and green lines indicate the complexes with the non-intercalated or intercalated drug bound, respectively. Dotted lines separate the DNA region from the different htopIB domains, as indicated by the labels.

To further characterize the main regions showing different flexibilities in the bound and unbound htopIB-DNA complexes, principal component analysis (PCA) was performed for each MD trajectory [31]. This technique allows for the isolation of the major fluctuations that contribute to the dynamics of each structure, identifying the principal 3 N directions along which the majority of the protein and DNA motion is defined. Atomic displacements, calculated for each Cα atom of htopIB and P atom of DNA along the first eigenvector, indicate that the unbound htopIB structure shows an overall higher degree of flexibility, mainly in the linker domain, in subdomain III, and in the C-terminal domain (Figure 7). Interestingly, the htopIB-DNA structure, complexed with the intercalated drug, shows a stronger rigidity within subdomain I that is not observed in the presence of the non-intercalated drug. The linker domain is the most flexible region of htopIB in both the unbound and bound systems, although in the unbound system this region shows about two-fold the flexibility observed in the two bound states. These data confirm the strongly correlated motions observed between the three main htopIB domains in the unbound complex DCCM (Figure 6A,B; upper-left triangles), and also validate the finding that the presence of the drug induces an evident structural rigidity in the protein, particularly in those residues directly facing the DNA region (residues 488–500, 532–535, 631–640, 713–722) (Figure 7). 

## 3. Discussion

Despite the fact that cancer knowledge has been growing in recent decades, the problem of cancerous disease persists, and these decades of scientific findings are still insufficient to solve it. The remarkable progresses made in cancer prevention, early detection, and treatment are still not sufficiently specific and effective. HtopIB plays essential roles in cell division by regulating all topological DNA stresses that arise during transcription or replication. For these reasons, htopoIB is an interesting target for cancer treatment. In this work we screened a library of antimalarial compounds, adopting a drug-repositioning screening, based on the evidence of *Plasmodium falciparum* topoisomerase homology with its human counterpart. In detail, we have characterized the effect of MMV024937 as a new drug, provided by the open-access drug bank MMV, on htopIB through experimental and computational approaches. To test MMV024937’s inhibition of htopIB activity, whether as a poison, in a CPT-like manner, or as a catalytic inhibitor such as erybraedin C, we performed in vitro assays. We demonstrated that MMV024937 affects htopIB as a catalytic inhibitor, and also in an irreversible manner (Figure 2B). 

The efficiency of MMV024937 as a potential anti-tumor drug was tested on cancer cell lines in comparison with TPT, a drug currently used in clinics for cancer treatment (Figure 4). We observed that the effect of MMV024937 is stronger than TPT at high concentrations, likely due to the fact that MMV024937 can act on pathways other than htopIB alone. Indeed, a previous work has demonstrated that MMV024937 is closely related to a series of human diacylglycerol acyltransferase-1 (DGAT-1) inhibitors [32]. DGAT-1 inhibition causes cancer cell death by inducing mitochondrial damage and elevated ROS formation, supporting the use of MMV024937 as a promising anti-tumor drug [33].

To better understand these results we also performed a computational analysis (Figure 5). Molecular docking and molecular dynamics simulations of MMV024937 modeled over the htopIB-DNA complex strongly support the observed inhibition effect, although they cannot provide information on its irreversibility. Computational results indicate that MMV024937 binds the htopIB-DNA complex sitting inside the catalytic site of the enzyme or intercalating within the DNA. Interaction analyses shows that the drug contacts several htopIB residues, including residues of the catalytic pentad such as Arg488, Lys532, Arg590, and Tyr723, as well as the neighbor DNA bases (Table 1 and Table 2). The persistent stability of the complexes and the strongly negative free energies of binding displayed by the drug during the 100 ns MD simulations provide a clear molecular explanation for the cleavage inhibition. The drug can exert its inhibitory activity, preventing DNA and htopIB contacts, by establishing a steric hindrance within the htopIB-DNA cleft and strong hydrophobic and electrostatic interactions with surrounding residues and DNA bases. Furthermore, in both the evaluated binding configurations, the presence of a single drug molecule can strongly influence the structural dynamics of htopIB-DNA structure. It is known that intra- and inter-domain communications are fundamental for htopIB’s catalytic activity and that DNA strand rotation is strongly dependent on the enzyme’s conformation and dynamics [34]. In particular, a different flexibility of the linker domain was shown to correlate with the rate of DNA religation reactions. Indeed, this region has a role in slowing the religation step, allowing the enzyme to remain associated to DNA for a larger number of cleavage/religation rounds, and it is also fundamental for DNA-controlled rotation [35,36]. During MD simulations we observed that the presence of the drug within the binding pocket almost completely abolished correlated motions of the complex, and strongly reduced htopIB linker flexibility. This is likely due to the strong interactions established by the drug with surrounding residues and DNA bases that decrease the protein–DNA structural movements and anchor the complex in an expected inactive state. Considering these results, we hypothesize that the structural stability induced by the drug should prevent the occurrence of the large conformational changes leading to the correct unwinding and relaxation of supercoiled DNA.

Despite MMV024937 may have additional targets beside htopIB; the evidence for relaxation and cleavage inhibition identifies this molecule as an interesting and valuable candidate in targeting htopIB in the context of cancer treatment.

## 4. Materials and Methods

### 4.1. Chemicals, Cells, Yeast Strains and Plasmids

MMV024937 were kindly provide by Malaria for Medicine Venture, Anti-FLAG M2 monoclonal affinity gel, FLAG peptide, Anti-FLAG M2 monoclonal antibodies, topotecan, 3-(4,5-Dimethyl-2-thiazolyl)-2,5-diphenyl-2H-tetrazolium bromide (MTT,) and dimethyl sulfoxide (DMSO) were purchased from MERCK (Darmstadt, Germany). In order to express the htopIB enzyme we used *Saccharomyces cerevisiae* Top1 null strain EKY3 (ura3–52, his3Δ200, leu2Δ1, trp1Δ63, top1::TRP1, MATα). Single copy plasmid YCpGAL-e- hTop1 was used to express the enzyme under a galactose-inducible promoter. The N-terminal sequence of the epitope-tagged construct YCp-GAL-e-, indicated as ‘e’, contains a FLAG sequence, DYKDDDY, and it is recognized by the M2 monoclonal antibody. The cloning reaction was transformed into XL10-Gold E. coli cells (Agilent Technologies, Santa Clara, CA, USA), and a positive clone was identified by sequencing the extracted plasmid DNA. Oligonucleotides for radioactive assays were purchased from Eurofins Genomics. [γ-32P] ATP was purchased from PERKIN Elmer (Waltham, MA, USA). 

Dulbecco’s modified Eagle’s medium high glucose, RPMI 1640 medium, fetal bovine serum (FBS), L-glutamine, penicillin/streptomycin, and non-essential amino acids were purchased from Euroclone (Pero, Italy). Complete media (CM) were supplemented with 10% FBS, 2 mM L-glutamine, 0.1 mg/mL streptomycin, and 100 U/mL penicillin. The ovarian cancer cell line SKOV-3 was purchased from Cell Biolabs, Inc. and maintained in DMEM-high glucose, CM supplemented with 1X non-essential amino acids; colorectal adenocarcinoma cell line, Caco-2, was maintained in RPMI 1640, CM; and non-small-cell-lung cancer cell line, A-549, was maintained in DMEM high glucose, CM. Caco-2 and A-549 cell lines were kindly provided by Dr. Giuseppe Sconocchia (Institute of Translational Pharmacology, CNR, Rome, Italy). The cells were tested for mycoplasma using the PCR detection Kit (Euroclone). The cells were kept in culture for a maximum of eight passages.

### 4.2. Protein Purification

In order to purify htopIB, the enzyme was cloned in a single copy plasmid YCpGAL under a galactose-inducible promoter. The purification was carried out by using the lithium acetate procedure, and the transformation was made in top1 null EKY3. Transformed cells were grown on SC-Uracil, with added 2% dextrose. Subsequently, the cells were diluted 1:100 in SC-Uracil with 2% raffinose until an optical density of A600 = 1 was reached, and then the transformed cells were induced with 2% galactose for 6 h. A washing with cold water occurred after the harvesting of the cells. In the next step the cells were resuspended in 2 mL/g cells using a buffer made up by 50 mM Tris/HCl, pH 7.4, 1 mM EDTA, 1 mM EGTA, 10% (*v/v*) glycerol, protease inhibitor cocktail, and supplemented with 10 mg/mL sodium bisulfite and 40 mg/mL sodium fluoride, 1 mM PMSF, and 1 mM DTT. The cells were disrupted by adding of 0.5 volume of 425–600 mm diameter glass beads, and by vortexing the solution for 30 s, alternating with 30 s on ice. In order to separate the glass beads from the supernatant, the solution was centrifuged at 12,000 rpm for 30 min. The column was then washed with TBS (50 mM Tris/HCl and 150 mM KCl, pH 7.4) and the ANTI-FLAG M2 affinity gel (MERCK, Darmstadt, Germany) was equilibrated. Several elutions of the protein were obtained by adding five columns volume of a solution containing the FLAG peptide, whereupon 500 µL of htopIB were supplemented with 40% glycerol and stored at −20 °C. In order to test the integrity of the protein, the fractions were resolved by SDS-PAGE and then shown through the immunoblot.

### 4.3. Relaxation Assay

The activity of the htopIB was assessed through the relaxation of negatively pBlueScript KSII (-) DNA. The reaction was carried out in a final volume of 30 µL containing a buffer composed of 20 mM Tris-HCl pH 7.5, 0.1 mM EDTA, 10 mM MgCl_2_, 50 µg/m acetylated bovine serum albumin, 150 mM KCl, and ddH_2_O. The reaction was stopped after 1 h incubation at 37 °C by adding 0.5% SDS stop dye. The samples were resolved in 1% agarose gel and in a running buffer containing 48 mM Tris, 45.5 mM boric acid, 1 mM EDTA. The enzyme’s ability to relax supercoiled DNA was visualized through a UV transilluminator after a gel staining in 0.5 µg/mL ethidium bromide and destaining in dH_2_O.

### 4.4. Cleavage Kinetics Using CL14/CP25 Oligonucleotide Substrate

In order to analyze the cleavage kinetics a CL14 (5′-GAAAAAAGACTTAG-3′) was radiolabeled with [γ-32P] ATP at its 5′ end through a 30 min incubation of 10 pmol of CL14 with a T4 kinase (New England Biolabs, Ipswich, MA, USA) buffer, 10 µL of [γ-32P] ATP and T4 kinase in a final volume of 50 µL at 37 °C. The radiolabeled oligonucleotide has been purified with MicroSpin g-25 (Amersham Bioscences, Amersham, United Kindom). The CL14 oligonucleotide contains a htopIB high affinity cleavage site. CL14 was annealed to a complementary oligonucleotide called CP25 (5′-TAAAAATTTTTCTAAGTCTTTTTTC-3′) that was phosphorylated with ATP at its 5′ end. The annealing between the oligonucleotides was carried out by incubating 10 pmol of CL14 with 20 pmol of CP25 in the presence of an Annealing Buffer at 95 °C for 5 min and then overnight at 4 °C.

The suicide cleavage reaction was carried out by incubating 20 nM of the duplex CL14/CL25 with htopIB in a buffer containing 10 mM Tris (pH 7.5), 5 mM MgCl_2_, 5 mM CaCl_2_, and 150 mM KCl at 25 °C in a final volume of 60 µL. After adding the enzyme, aliquots of 5 µL were removed at different times and the reactions were stopped by adding 0.5% SDS. After a precipitation with ethanol, the samples were resuspended in 5 µL of 1 mg/mL of trypsin and incubated at 37 °C for 1 h. The samples were analyzed by electrophoresis on denaturing polyacrylamide gel (7 M urea, 20% Acrylamide) in TBE running buffer (48 mM Tris, 45.5 mM Boric Acid and 1 mM EDTA). The percentage of cleaved substrate (CL1) has been evaluated.

### 4.5. Cell Viability Assay

To evaluate cell viability 1 × 10^4^ tumor cells (Caco-2, A-549 and SKOV-3) were seeded in a 96-well plate for 24 h at 37 °C, 5% CO_2,_ and 95% humidity. The day after, each cell line was treated with a different amount of MMV024937 or topotecan, ranging from 12.5 µM to 100 µM. As a control, the cells were treated with the same amount of DMSO as well. The plates were then incubated for 48 h at 37 °C under 5% CO_2_. After the incubation, the medium was removed and 200 μL of fresh CM supplemented with MTT reagent at final concentration of 0.5 mg/mL was added and incubated again for 4 h at 37 °C and 5% CO_2_. The reagent was then replaced with 100 μL per well of DMSO and incubated at room temperature for 15 min on a shaking plate, covered from light. The absorbance was measured within an hour at 570 nm using a microplate reader; the data was analyzed with GraphPad Prism.

### 4.6. Molecular Docking Simulations

A crystal structure of the DNA-bound htopIB, lacking the N-terminal domain (residues 1–200), was obtained from the PDB database (PDB ID: 1T8I) [27] and used as a receptor for molecular docking simulations. The gap between nucleotides dT10 and dG11 was repaired. The tleap module of the AmberTools19 program [37], the parmbsc1 force field [38], and the Chimera software [28], were used to reconstruct the two nucleotides and the missing phosphodiester bond. A CPT molecule, originally bound to the crystal structure, was removed from the complex. The structure of the drug MMV024937 was retrieved from the PubChem compound database (PubChem CID: 44528432) [39]. Receptor and drug structure files were converted into pdbqt format using the prepare_receptor4.py and prepare_ligand4.py tools of the AutoDockTools4 program [40]. Protein-ligand molecular docking simulations were performed using the AutoDock Vina program [41]. Ten molecular docking simulations, each including ten docking runs, were performed using a box of size x = 27.4 Å; y = 27.4 Å; z = 23.3 Å, centered over the htopIB-DNA binding site. To increase the accuracy of binding pose estimation, 10 receptor residue side chains around the binding site were regarded as flexible (Arg364, Arg488, Lys532, Asp533, Ile535, Arg590, Asn631, His632, Thr718 and Tyr723). The final interaction energies were calculated as an average over the 10 replicas of the docking simulations. Interaction analyses on the best binding poses obtained were performed using the Ligplot+ software [29].

### 4.7. Classical Molecular Dynamics Simulations and Trajectory Analysis

Topologies and coordinates files for the two best complexes obtained from molecular docking simulations and for the unbound htopIB-DNA structure (PDB ID: 1T8I) [27], simulated as reference, were generated using the tleap module of the AmberTools19 program [37]. The AMBER ff19SB [42] and parmbsc1 [38] force fields were used to parametrize the htopIB and DNA, while ligand parameters were generated using the antechamber module of the AmberTools19 program [37] and the general Amber force field [43]. Each complex were inserted in a box of TIP3P water molecules [44] and 0.15 mol/L of NaCl, setting a minimum distance of 12.0 Å from the box sides. To remove unfavorable interactions, four minimization cycles were performed for the three structures, each composed of 500 steps of the steepest descent minimization, followed by 1500 steps of conjugated gradient. A starting restraint of 20.0 kcal·mol −1·Å −2 was imposed on the protein, DNA, and ligand atoms; it was then slowly reduced and removed in the last minimization cycle. Systems temperature was gradually increased from 0 to 300 K in an NVT ensemble, using the Langevin thermostat [45], over a period of 2.0 ns. A starting restraint of 0.5 kcal·mol −1·Å −2 was imposed on the protein, DNA, and ligand atoms and then gradually decreased to slowly relax the system. Systems were then simulated in an isobaric-isothermal (NPT) ensemble for 2.0 ns using the Langevin barostat [46], imposing a pressure of 1.0 atm and maintaining the temperature to 300 K. The SHAKE algorithm [47] was used to constrain covalent bonds involving hydrogen atoms. Production runs of 100 ns were generated for each system using the pmemd.cuda module of the AMBER16 software [48] and a timestep of 2.0 fs. System coordinates were written every 1000 steps. Long-range interactions werecalculated using the PME method [49], while a cut-off of 9.0 Å was imposed for short-range interactions. 

### 4.8. Trajectory Analysis

Principal component analysis (PCA) [30] has been performed for each trajectory on Cα atoms of the htopIB and P atoms of DNA using the GROMACS 2019 program [50]. This analysis is based on the diagonalization of a covariance matrix, generated for each trajectory using the covar module of GROMACS [50], and built from the atomic fluctuations of Cα atoms and P atoms after the removal of translational and rotational movements. PCA analyses showed that, for all three systems, the main motion is dispersed over 1821 eigenvectors, but 80% of the total protein and DNA motion can be described by the first 6 eigenvectors for the unbound system and by the first 17 and 16 eigenvectors for the non-intercalated and intercalated systems, respectively. In particular, the first eigenvector, with the largest eigenvalue, accounted for 58% of the total motion for the unbound htopIB-DNA complex and for 32% and 34% of the total motion for the htopIB-DNA complexes bound to the non-intercalated and intercalated drug, respectively. Dynamic cross-correlation maps (DCCMs) were computed from the covariance matrices generated for each trajectory using code written in-house. Plots were realized using the matplotlib Python 3 library. Molecular mechanics/generalized Born and surface area continuum solvation (MM/GBSA) analyses [51] were performed over the last 50 ns of each trajectory, using the MMPBSA.py.MPI program implemented in the AMBER16 software [48] on three nodes of the of ENEA HPC cluster CRESCO6 [52], setting the ionic strength to 0.15 M. Per-residue decomposition analysis was performed on DNA bases surrounding the drug (nucleotides dT10, dG11, dG12, dA13, dC34, dA35, dA36, dG37) and on htopIB active site residues (residues Arg488, Lys532, Arg590, His632, Tyr723).

## Figures and Tables

**Table 1:** Results of the MM/GBSA analyses of the MD trajectories of the htopIB-DNA complex with the drug in non-intercalated (Figure 5A) or intercalated (Figure 5C) configuration.

Drug Binding Configuration	VdW (kcal/mol)	Electrostatic (kcal/mol)	Interaction Energy (kcal/mol)
non-intercalated	−63.7 ± 2.8	−14.4 ± 9.5	−49.0 ± 3.4
intercalated	−67.1 ± 2.9	−64.0 ± 12.3	−55.1 ± 4.0

**Table 2:** MM/GBSA per-nucleotide/residue decomposition analyses performed for the MD trajectories of the htopIB-DNA complexes bound to the drug in non-intercalated (Figure 5A) and intercalated (Figure 5C) configurations. Interaction energies were evaluated between the drug and surrounding DNA bases or htopIB active site residues.

Nucleotide/Residue	Non-Intercalated Drug Binding Energy (kcal/mol)	Intercalated Drug Binding Energy (kcal/mol)
dT10	−2.0 ± 0.5	−3.0 ± 1.8
dG11	−0.1 ± 0.1	−5.8 ± 0.9
dG12	−3.0 ± 1.1	−0.4 ± 0.2
dA13	−2.1 ± 1.7	+0.1 ± 0.0
dC34	+0.1 ± 0.0	−4.4 ± 1.0
dA35	0.0 ± 0.0	−6.1 ± 1.0
dA36	−0.1 ± 0.1	−0.7 ± 0.2
dG37	−0.7 ± 0.4	−0.2 ± 0.2
Arg488	−0.8 ± 0.6	−0.9 ± 0.5
Lys532	−2.7 ± 0.6	−0.8 ±1.0
Arg590	+0.1 ± 0.4	−0.1 ± 0.1
His632	−1.8 ± 0.4	−0.2 ± 0.2
Tyr723	−1.3 ± 0.5	−0.3 ± 0.2
